# The basic reproductive ratio of the 2022 outbreak of the monkey pox virus disease for the United Kingdom, Canada, Brazil, the United Arab Emirates, and Nigeria

**DOI:** 10.1186/s43088-022-00316-x

**Published:** 2022-11-07

**Authors:** Marwan Al-Raeei

**Affiliations:** Faculty of Sciences, Damascus University, Damascus, Syrian Arab Republic

**Keywords:** Basic reproductive ratio, Fitting, SIRD model, Basic reproduction number, SIR, BRRMPD, Monkey pox virus disease, Disease spreading, Pandemic, WHO, MSM

## Abstract

**Background:**

A recent outbreak of the monkey pox virus disease (MPVD) started to spread over the world before the second half of the 2022 year. This outbreak of the monkey pox virus disease is known as the 2022 outbreak of the monkey pox virus disease. The monkey pox virus disease is a type of the pox disease similar to the human one. This disease is an endemic in some African countries; however, a new spreading of this disease started to appear in other countries, such as the Spain, brazil, Greece, the United Kingdom, and Portugal, Australia, and the USA. As of the end of September 2022, the MPVD spread over than 107 countries over the world.

**Results:**

This study focuses on the employing of the simplest model of the diseases forecasting which is SIRD model for the finding of the basic reproductive ratio of the monkey pox virus disease in multiple countries over the world where the disease spreads. The model takes into accounts the number of the susceptible people, the number of the infectious people, the number of the recovered people, and the number of the deceased people. Based on the results of the SIRD model coefficients, we find that the basic reproductive ratio values of the recent spreading of the monkey pox virus disease are 1.3274 for the United Kingdom where the first case of the disease was recorded, 1.0714 for the United Arab Emirates, 1.0866 for Nigeria, 1.5589 for Brazil, and 1.3610 for Canada.

**Conclusions:**

We find that the average value of the basic reproductive ratio of the 2022 outbreak of the monkey pox virus disease is about 1.2809. This important result of our calculations predicts that the 2022 outbreak of the monkey pox virus disease is turned into pandemic over the world. The things which confirm this result, based on our calculations, are the values of the basic reproductive ratio of the 2022 outbreak of the disease in the considered countries from multiple continents where all the values of the basic reproductive ratio are bigger than one. From this point, the counties over the world must apply multiple procedures for limiting the spreading of the monkey pox virus disease.

**Graphical Abstract:**

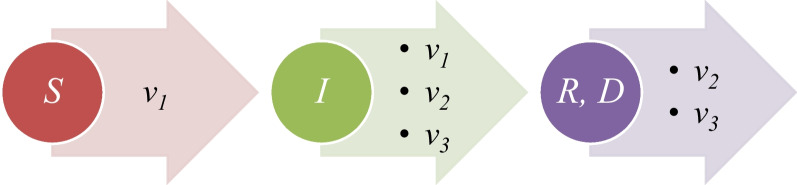

## Background

At the end of the first half of 2022, new cases of the viral virus, namely the monkey pox virus disease (MPVD), started to appear in multiple countries over the world. The recent spread of the monkey pox virus disease is known as the 2022 outbreak of the MPVD. The first case of the 2022 outbreak of the monkey pox virus disease was recorded in May 2022 in the United Kingdom. The 2022 outbreak of the monkey pox virus disease spread in lots of countries over the world. For example and as of the end of September 2022, in some of the European continent, the disease appeared in Belgium with 744 confirmed cases [1], in Austria with 300 confirmed cases [2], in Bulgaria with six confirmed cases [2], in Denmark with 183 confirmed cases [3], in Hungary with 75 confirmed cases [2], Sweden with 179 confirmed cases [4], in Switzerland with 502 confirmed cases [5], in France with 3898 confirmed cases [6], in Italy with 837 confirmed cases [7], in Germany with 3570 confirmed cases [8], and in the United Kingdom with 3585 [9], whereas we have mentioned, the first case of the 2022 monkey pox virus disease was recorded. Besides, and as of end of September 2022, the European country with the highest cases of the 2022 of the monkey pox virus disease is Spain with 6749 confirmed cases [10]. Also up to the same date, the monkey pox virus disease appeared in some of the Latin American continent countries, and for example, the pandemic appeared in Argentina with 326 confirmed cases [11], in Colombia with 1260 confirmed cases [12], and in Brazil with 7019 confirmed cases [13], which is the highest country of the Latin America countries with cases of the monkey pox virus disease as of end of September 2022, while in Australia, the pandemic appeared with 132 confirmed cases [14]. In the North American continent, the pandemic appeared with 1363 confirmed cases in Canada [15–18], and 23,893 confirmed cases in the USA [2, 19]. As of the end of September 2022, the number of cases recorded in the USA was the highest number of the total confirmed cases over the world. In Asia in the far east, the monkey pox virus pandemic appeared with one confirmed case in Hong Kong [20], and one confirmed case in China [21]. Also in Asia in the Middle east, the monkey pox virus pandemic appeared with 16 confirmed cases in the United Arab Emirates based on the governmental data [22], in Lebanon with 11 confirmed cases [2], and with eight confirmed cases in Saudi Arabia [23]. Lots of indicators can be employed for the discussing the future of the recent spreading of the monkey pox disease such as the basic reproductive ratio of the monkey pox virus disease (BRRMPD), the force of infection of the monkey pox virus disease (FIMPVD), and the incubation period of the monkey pox virus disease (IPMPVD). In this study, we use the simple SIRD model, which takes its name from the number of susceptible people, the number of the infectious people, the number of the recovered, and the number of the deceased people [24–26], for finding initial values of the basic reproductive ratio of the 2022 outbreak of the monkey pox virus disease in multiple countries from multiple continents over the world.

**Fig. 1:**
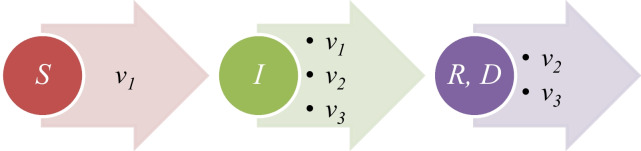
Schematic graph of the SIRD model

The MPVD is not a new, where this disease is an endemic in some of the African countries. However, in the recent months, this disease started to appear in new countries over the world. Multiple studies about the 2022 outbreak of the monkey pox virus disease were discussed. For example, Hemati et al. proposed useful study of the using of procedures which were taken of the spreading of the corona virus disease 2019 [27]. de Jonge et al. found in Netherland that there is deoxyribonucleic acid (DNA) of the monkey pox virus in the wastewater samples [28]. Rao et al. study the detection of the monkey pox virus disease of traveller from Africa [29]. Vaughan et al. reported the 2022 outbreak of the monkey pox virus disease over the World Health Organization European countries for a specific time [30]. Meaney Delman et al. [31], Long et al. [32], Lee and Morling [33], and Matias et al. [34] showed some procedures for the limitation of monkey pox virus disease. Sah et al. showed the monkey pox virus disease in Asia [35]. Lozada Martinez discussed the update of the monkey pox virus disease in the Latin American continent [36]. Pomar et al. showed the status of the monkey pox virus disease during the pregnancy [37]. Mungmunpuntipantip and Wiwanitkit discussed the monkey pox virus disease cases with some cases which had human immunodeficiency virus (HIV) disease [38]. Chakraborty et al. showed the vaccines of the monkey pox virus disease [39]. Subedi and Acharya showed the outbreak of the monkey pox virus disease in Nepal [40]. Lapa et al. found and isolated the monkey pox virus from a semen sample [41]. Also, based on the World Health Organization (WHO) [42], and the centers for disease control and prevention [2], more than 95% of the total confirmed cases were recorded in people who have sex men to men. Another significant study about the monkey pox virus disease talked about airborne transmission of the monkey pox virus disease [43]. As we have mentioned, in this study, we used the SIRD model with the fitting techniques for finding the basic reproductive ratio of the 2022 outbreak of the monkey pox virus disease. The SIRD model contains four different equations: the first equation is a type of the nonlinear equation; this equation gives the rate of the susceptible cases as a function to the product of the susceptible cases, and the infectious cases, and the parameter in this equation is coefficient of infection which is *ν*_1_. The second equation is also a type of the nonlinear equations, and this equation gives the rate of the infectious cases as a function to the product of the infectious cases itself, and the susceptible cases, with also another term as function to the infectious cases itself. The second equation has three different parameters, and these parameters are the coefficient of infection which is *ν*_1_, the coefficient of recovery which is *ν*_2_, and the coefficient of the mortality *ν*_3_. The third equation of the model gives the recovery rate as a function to the infectious cases, and the parameter of this equation is the coefficient of the recovery *ν*_2_. The fourth and the last equation of the epidemic model, which we use, gives the mortality rate as a function to the infectious cases, and the parameter of this equation is the coefficient of the mortality *ν*_3_. In Fig. 1, we illustrate that a schematic graph represents the SIRD epidemic model. In the following, in the second section of the article, we illustrate the method used for finding the values of basic reproductive ratio of the 2022 outbreak of the monkey pox virus disease. In the third section of this article, we illustrate the results which we found of the basic reproductive ratio of the 2022 outbreak of the MPVD for five counties from multiple continents over the world, while in the last section, we illustrate the conclusion of the study.

## Methods

We collect the confirmed recorded cases of the 2022 outbreak of the monkey pox virus disease for multiple countries over the world. Then, we use the SIRD epidemic model and the simple fitting method based on the numerical simulation techniques using MATLAB software, for finding the basic reproductive ratio of the 2022 outbreak of the monkey pox virus disease. The basic reproductive ratio of the disease based on the equations of the epidemic model can be derived using multiple methods. For instance, in this work, we illustrate the definition of the basic reproductive ratio of the SIRD epidemic model based on the Jacobean method. For the derivation of the basic reproductive ratio of the SIRD model, we write the Jacobean of the SIRD model as follows:

1 $${\rm Jac} \equiv \left[ {\begin{array}{*{20}c} { - N^{ - 1} \nu_{1} I} &\quad { - N^{ - 1} \nu_{1} S} &\quad 0 &\quad 0 \\ {N^{ - 1} \nu_{1} I} &\quad { - \nu_{2} - \nu_{2} + N^{ - 1} \nu_{1} S} &\quad 0 &\quad 0 \\ 0 &\quad {\nu_{2} } &\quad 0 &\quad 0 \\ 0 &\quad {\nu_{3} } &\quad 0 &\quad 0 \\ \end{array} } \right]$$

where *S* represents the total number of the susceptible people, *I* represents the total number of the infectious people, *R* represents the total the number of the recovered, and *D* represents the total number of the deceased people, while *N* is the total number of the population in a specific society or a specific country. We can write the equilibrium case of the epidemic based on the Jacobean of the SIRD model as follows:

2 $${\rm Jac}_{0} = \left[ {\begin{array}{*{20}c} 0 &\quad { - \nu_{1} } &\quad 0 &\quad 0 \\ 0 &\quad {\nu_{1} - (\nu_{2} + \nu_{3} )} &\quad 0 &\quad 0 \\ 0 &\quad {\alpha_{2} } &\quad 0 &\quad 0 \\ 0 &\quad {\alpha_{3} } &\quad 0 &\quad 0 \\ \end{array} } \right]$$

where 0 in Jac_0_ represents the equilibrium case of the epidemic. The next step is finding the eigenvalues of the equilibrium Jacobean of the SIRD model, which is found from the following well-known equation:

3 $$\left| {\alpha \sigma_{4} - {\rm Jac}_{0} } \right| = 0$$

where *α* represents the eigenvalues of the Jacobean of the SIRD model, and *σ*_4_ represents the identity matrix which is given as follows:

4 $$\sigma_{4} = \left[ {\begin{array}{*{20}c} 1 &\quad 0 &\quad 0 &\quad 0 \\ 0 &\quad 1 &\quad 0 &\quad 0 \\ 0 &\quad 0 &\quad 1 &\quad 0 \\ 0 &\quad 0 &\quad 0 &\quad 1 \\ \end{array} } \right]$$

Equation 3 gives us the following fourth degree equation of the eigenvalues:

5 $$\nu_{1} \alpha^{3} - \nu_{2} \alpha^{3} - \alpha^{4} - \nu_{3} \alpha^{3} = 0$$

We solve the previous algebraic equation for the eigenvalues, which gives three zeros solutions:

6 $$\alpha_{1} = 0$$

7 $$\alpha_{2} = 0$$

8 $$\alpha_{3} = 0$$

and one nonzero solution:

9 $$\alpha_{4} = \nu_{1} - \nu_{2} - \nu_{3}$$

The nonzero solution gives us the stable and the non-stable equilibrium case of the epidemic as follows:

10a $$\nu_{1} - \nu_{2} - \nu_{3} < 0$$

10b $$\nu_{1} - \nu_{2} - \nu_{3} > 0$$

which gives us the basic reproductive ratio of the SIRD model as follows:

11 $$R_{0} = \frac{{\nu_{1} }}{{\nu_{2} + \nu_{3} }}$$

**Fig. 2:**
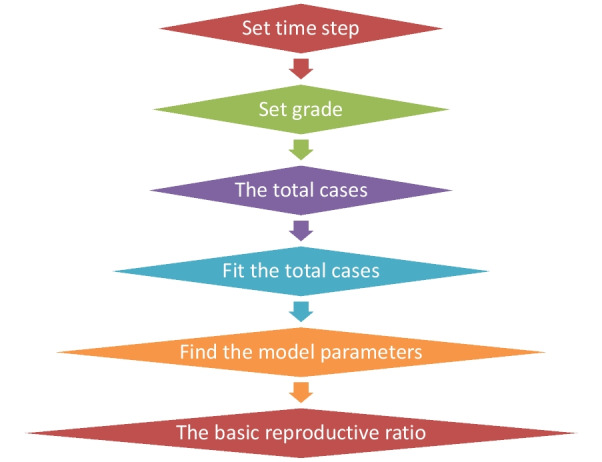
A schematic graph of the numerical simulation algorithm which we used for the fitting of the SIRD model of MPVD, and finding the basic reproductive ratio of the MPVD

As we see from the previous equation, by finding the three parameters of the SIRD model, we can find the basic reproductive ratio of the 2022 outbreak of the monkey pox virus disease. As we can see from the last equation, if the disease has very low mortality rates, the mortality parameter of the SIRD model can be neglected, and we return to the basic reproductive ratio of the simple case without mortality. We fit the collected cases of the monkey pox virus disease in each country of the considered countries for finding the parameters of the SIRD model for the monkey pox virus disease. After that we use Eq. 11 for finding the basic reproductive ratio of the 2022 outbreak of the monkey pox virus disease. In Fig. 2, we illustrate a schematic graph which represents the algorithm which we use for finding the basic reproductive ratio of the 2022 outbreak of the monkey pox virus disease.

## Results

We used the collected data of the monkey pox virus disease in five countries where we chose one country from each continent. As an example of the North American continent countries, we chose Canada for finding the basic reproductive ratio of the 2022 outbreak of the monkey pox virus disease. We found the coefficients of the SIRD model of the 2022 outbreak of the monkey pox virus disease for Canada, and we put the results of the calculations in Table 1 with the days^−1^ unit, where the mortality coefficient is very small and near zero. As an example of the Latin American continent countries, we chose Brazil for finding the basic reproductive ratio of the 2022 outbreak of the monkey pox virus disease. Brazil is the third country over the world with the monkey pox virus disease cases as of the end of September 2022. We found the coefficients of the SIRD model of the 2022 outbreak of the monkey pox virus disease for Brazil, and we put the results of the calculations in Table 1 with the days^−1^ unit, where the mortality coefficient, as in Canada, is very small and near zero. As an example of the African continent countries, we chose Nigeria for finding the basic reproductive ratio of the 2022 outbreak of the monkey pox virus disease. We found the coefficients of the SIRD model of the 2022 outbreak of the monkey pox virus disease for Nigeria, and we put the results of the calculations in Table 1 with the days^−1^ unit, where the mortality coefficient is small and equal to 0.0016 days^−1^, and it is not very small, and this returns to there are four death cases of the disease in Nigeria as of the end of September 2022. As an example of the European continent countries, we chose the United Kingdom for finding the basic reproductive ratio of the 2022 outbreak of the monkey pox virus disease. The United Kingdom is the country where the first case of the recent spreading of the disease was recorded. We found the coefficients of the SIRD model of the 2022 outbreak of the monkey pox virus disease for the United Kingdom, and we put the results of the calculations in Table 1 with the days^−1^ unit, whereas in Canada, the mortality coefficient is very small and near zero. Finally, and as an example of the Asian continent countries, we chose the United Arab Emirates for finding the basic reproductive ratio of the 2022 outbreak of the monkey pox virus disease. The United Arab Country is one of the Arabic world countries. We found the coefficients of the SIRD model of the 2022 outbreak of the monkey pox virus disease for the United Arab Emirates, and we put the results of the calculations in Table 1 with the days^−1^ unit, whereas in Canada, the mortality coefficient is very small and near zero.

**Table 1:** Coefficients of the SIRD model of the monkey pox pandemic for Brazil, Canada, Nigeria, the United Arab Emirates, and the United Kingdom

	$\nu_{1} (d^{ - 1} )$	$\nu_{2} (d^{ - 1} )$
Canada	0.0349	0.0256
The United Arab Emirates	0.0313	0.0292
Brazil	0.0540	0.0347
Nigeria	0.0442	0.0391
The United Kingdom	0.0551	0.0415

Depending on the previous calculations of the coefficients of the SIRD model, we found the values of the basic reproductive ratio of the 2022 outbreak of the monkey pox virus disease for the five countries: Brazil, Canada, Nigeria, the United Arab Emirates, and the United Kingdom. We put the results in Table 2.

**Table 2:** Basic reproductive ratio of the 2022 outbreak of the monkey pox virus disease for Brazil, Canada, Nigeria, the United Arab Emirates, and the United Kingdom

	Canada	The United Arab Emirates	Brazil	Nigeria	The United kingdom
The basic reproductive ratio	1.3610	1.0714	1.5589	1.0866	1.3274

As shown in Table 2, the average value of the basic reproductive ratio of the 2022 outbreak of the monkey pox virus disease equals to 1.2809 which means that the disease is a pandemic.

## Conclusions

In this work, we discussed the initial basic reproductive ratio of the 2022 outbreak of the monkey pox virus disease, where this disease started to appear in multiple countries in all of the continents, especially, in the European countries. The simple model of the spreading of the disease was applied which is the SIRD model. The values of the basic reproductive ratio of the 2022 outbreak of the monkey pox virus disease were found, where the values of the coefficient of the recovery, the coefficient of the mortality, and the coefficient of the infection were calculated for five countries from different continents over the world. The five considered countries are Canada as a country of the North American countries, the United Kingdom as a country of the European countries, Nigeria as a country of the African countries, Brazil as a country of the Latin American countries, and the United Arab Emirates as a country of the Asian countries. We found that the basic reproductive ratio of the MPVD is 1.3274 for the United Kingdom, where the first case of the disease was recorded, 1.0714 for the United Arab Emirates, 1.0866 for Nigeria, 1.5589 for Brazil, and 1.3610 for Canada.

We found that the average value of the basic reproductive ratio of the 2022 outbreak of the monkey pox virus disease equals to 1.2809 which means that the MPVD have returned into a pandemic in its 2022 outbreak. This means that multiple procedures for limiting of the spreading of the disease must be applied. The method which we applied in this work has no limitations of applying in lots of countries for the study of the forecasting of the 2022 outbreak of the monkey pox virus disease using the basic reproductive ratio of the disease.

## Data Availability

The author confirms that the data are available for non-commercial using.

## Abbreviations

MPVD: Monkey pox virus disease
FIMPVD: Force of infection of the monkey pox virus disease
IPMPVD: Incubation period of the monkey pox virus disease
SIRD: Susceptible people, infectious people, recovered people, and deceased people
MSM: Men sex to men
DNA: Deoxyribonucleic acid
HIV: Human immunodeficiency virus
BRRMPVD: Basic reproductive ratio of the monkey pox virus disease
